# CyKILR splice variants: A double-edged sword in non-small cell lung cancer

**DOI:** 10.1016/j.omtn.2025.102538

**Published:** 2025-04-19

**Authors:** Muhammad Riaz Khan, Benoit Laurent

**Affiliations:** 1 Research Center on Aging, Centre Intégré Universitaire de Santé et Services Sociaux de l'Estrie-Centre Hospitalier Universitaire de Sherbrooke, Sherbrooke, QC, Canada; 2 Department of Biochemistry and Functional Genomics, Faculty of Medicine and Health Sciences, Université de Sherbrooke, Sherbrooke, QC, Canada

## Main text

Xie et al. recently made the exciting discovery that a novel long non-coding RNA (lncRNA) (ENSG00000267053), termed cyclin-dependent kinase inhibitor 2A-regulated lncRNA (CyKILR), produces through alternative splicing multiple variants.^1^ Alternative splicing is a fundamental biological process that enhances the variety of protein-coding genes and lncRNAs, thereby augmenting transcriptome complexity and functional diversity. This mechanism enables a single gene containing multiple exons to produce various transcripts, each potentially with distinct functions. In this context, Xie and colleagues demonstrated that CyKILR gene produces two distinct splice variants with opposing functional roles in the tumorigenesis of non-small cell lung cancer (NSCLC).^1^ Their findings underscore the critical need to investigate the functional and mechanistic roles of previously overlooked lncRNA splice variants, which may have significant implications for our comprehension of cancer biology and the development of innovative therapeutic approaches.

Long noncoding RNA is a class of RNA molecules defined as transcripts exceeding 200 nucleotides in length and lacking potential open reading frames for protein translation. A growing number of lncRNA genes have been identified across various genomes and emerged as a critical component of the mammalian transcriptome. Similar to protein-coding genes, lncRNA genes typically contain multiple exons and introns.^2^ Hence, lncRNA genes undergo various RNA maturation processes, including capping, polyadenylation, and alternative splicing. These processes are essential for producing mature and functional lncRNA transcripts that can perform diverse regulatory roles within the cell. For instance, lncRNAs can act as epigenetic modifiers, transcriptional activators or repressors, protein scaffolds, and molecular sponges for microRNAs and other RNA types.^3^ Alternative splicing of lncRNA genes generates functional diversity essential for precise gene expression regulation and cellular process control.^4^ This diversity stems from the capacity of a single lncRNA gene to produce multiple transcript variants that could serve unique functions in normal and pathological conditions.^5^ To illustrate this concept, Xie et al. demonstrated that CyKILR lncRNA has two splice variants that differentially contribute to the complex landscape of NSCLC tumorigenesis.^1^

The CDKN2A gene encodes the tumor suppressors p16(INK4A) and p14(ARF) and is often inactivated in NSCLC. In this study by Xie and colleagues, transcriptomic analyses were conducted on twelve NSCLC cell lines with varying CDKN2A statuses (wild type vs. inactive/mutant). This analysis identified CyKILR as a significantly differentially expressed lncRNA, suggesting its potential role in NSCLC development. Further analyses revealed that CyKILR expression was notably high in cell lines that also had wild-type STK11 genes, suggesting that the concurrent presence of active CDKN2A and STK11 genes is associated with CyKILR expression in NSCLC. These findings imply a potential regulatory relationship between these tumor suppressor genes and CyKILR, warranting further exploration into their combined roles in NSCLC pathogenesis.

**Figure 1:**
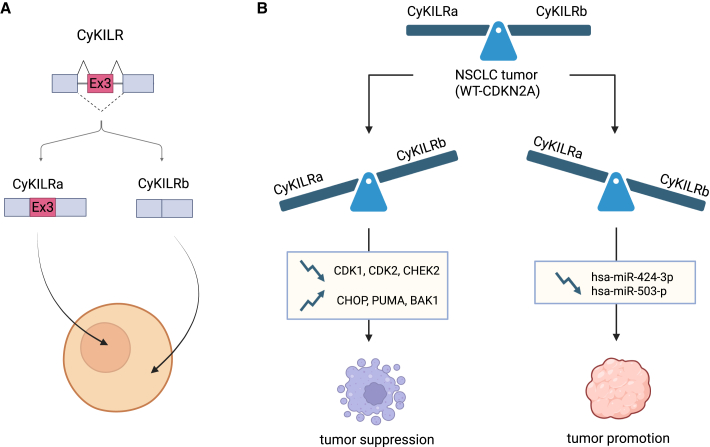
Regulation and functional impact of CyKILR splice variants in NSCLC (A) Alternative splicing of CyKILR. CyKILR undergoes alternative splicing, generating two major splice variants: CyKILRa, which retains exon 3 (Ex3), and CyKILRb, which lacks exon 3. CyKILRa localizes predominantly to the nucleus, while CyKILRb functions elsewhere in the cell. (B) Functional roles of CyKILR variants in NSCLC. In NSCLC tumors with wild type (WT) CDKN2A, the balance between CyKILRa and CyKILRb influences tumor progression. CyKILRa promotes tumor suppression by downregulating genes involved in cell proliferation (CDK1, CDK2, and CHEK2) and upregulating apoptotic genes (CHOP, PUMA, and BAK1). Conversely, CyKILRb contributes to tumor promotion by downregulating tumor-suppressive microRNAs (hsa-miR-424-3p and hsa-miR-503-3p).

The researchers then discovered that CyKILR undergoes alternative splicing, resulting in two distinct variants: CyKILRa containing exon 3 and CyKILRb excluding exon 3. CyKILRa contains a nuclear localization signal within exon 3 and hence is predominantly found in the nucleus, while CyKILRb is localized to the cytoplasm due to the absence of exon 3 (Figure 1A). This differential localization underpins the divergent functional roles of the two variants. By its nuclear presence, CyKILRa likely influences gene expression through its interactions with chromatin, and epigenetic factors, consistent with the known functions of nuclear lncRNAs. Transcriptomic analyses revealed that depletion of variant CyKILRa suppresses apoptotic factors and induces cell cycle-related growth factors, demonstrating that CyKILRa acts as a tumor suppressor. Conversely, the cytoplasmic localization of CyKILRb suggests a potential role in post-transcriptional regulation, possibly acting as a molecular sponge for cytosolic tumor-suppressive microRNAs. Indeed, reducing CyKILRb expression elevates levels of tumor-suppressing microRNAs, such as hsa-miR-424-3p and hsa-miR-503-3p, suggesting that CyKILRb supports tumor growth (Figure 1B).

The study further elucidates the functional implications of CyKILR splice variants by showing that the downregulation of CyKILRb in NSCLC cell lines with active STK11 and CDKN2A genes leads to a significant reduction in clonogenic survival and tumor growth. This finding underscores the oncogenic nature of CyKILRb and suggests that the ratio of CyKILRb/CyKILRa variants is critical, with a shift toward CyKILRa potentially inhibiting tumor cell growth. Surprisingly, the downregulation of both CDKN2A and STK11-derived mRNAs, individually or concomitantly, did not consistently alter total levels of CyKILR in NSCLC cell lines. Interestingly, simultaneous downregulation of CDKN2A and STK11 dramatically increased the ratio of CyKILRb/CyKILRa variants, suggesting that these gene transcripts promote CyKILR exon 3 inclusion. However, the exact mechanisms by which CDKN2A and STK11 modulate the splicing machinery to favor the inclusion of exon 3 remain unclear and require further investigation.

The study by Xie and colleagues provides compelling evidence for the distinct functions of CyKILR splice variants in NSCLC. However, several questions remain to be addressed regarding the precise molecular mechanisms underlying these functions. It is unclear whether the tumor-suppressive role of CyKILRa is solely due to the inclusion of exon 3 or if other factors, such as the lncRNA’s secondary structure or RNA modifications, contribute to its function. Additionally, the study does not fully elucidate how the loss of exon 3 impacts the lncRNA localization and function beyond the nuclear-cytoplasmic dichotomy. Addressing these questions is essential for a comprehensive understanding of CyKILR’s role in NSCLC.

Moreover, the study underscores the necessity for further research to validate these findings in larger cohorts and to explore the therapeutic potential of targeting CyKILR splice variants. The use of antisense oligonucleotides (ASOs) to modulate alternative splicing events—either by enhancing CyKILRa expression or inhibiting CyKILRb—presents a promising avenue for future interventions. ASOs have demonstrated efficacy in altering splicing patterns, thereby producing therapeutic gene products in various diseases. However, the intricate nature of lncRNA biology and their context-dependent functions necessitate a cautious approach. Future studies should focus on elucidating the key downstream targets and pathways influenced by CyKILR, as well as the broader genomic and epigenetic contexts in which it operates, to fully harness its therapeutic potential in NSCLC.

In conclusion, Xie et al.’s groundbreaking research has unveiled the pivotal roles of CyKILR splice variants in NSCLC, highlighting the profound impact of lncRNAs on cancer biology. The identification of CyKILRa and CyKILRb variants, as well as the compelling evidence of their distinct functions not only deepen our understanding of lncRNA involvement in tumorigenesis but also open new avenues for therapeutic interventions. Targeting specific lncRNA splice variants, such as modulating CyKILRa and CyKILRb, could revolutionize RNA-based cancer therapies. However, future studies should focus on elucidating the precise molecular mechanisms governing lncRNA splicing and exploring the therapeutic potential of lncRNA splice variant modulation in NSCLC and other malignancies.

## Declaration of interests

The authors declare no competing interests.
